# Functional neurological disorder: Practical management

**DOI:** 10.1016/j.neurot.2025.e00612

**Published:** 2025-05-20

**Authors:** Barbara A. Dworetzky, Gaston Baslet

**Affiliations:** a Brigham and Women's Hospital, Mass General Brigham, Department of Neurology, Harvard Medical School, Boston, USA; b Bruce W. Carter Department of Veterans Affairs Medical Center, Miami, FL, USA; c Harvard Medical School, Boston, MA, USA

**Keywords:** Functional neurological disorder, FND, Dissociative, Psychogenic, Conversion

## Abstract

Functional Neurological Disorder (FND) is a common and disabling condition seen by nearly every clinician in nearly every clinical setting. There are multiple subtypes with seizure and motor/movement being the most common. There is high health care utilization and costs, and many patients have a chronic course and remain disabled. It is clear from research over the past two decades that abnormalities in brain network activity are implicated in the pathophysiology of FND. Diagnosis requires positive criteria and knowing how to obtain a good history and avoid common pitfalls. There are evidence-based treatments and expert consensus recommendations. A multidisciplinary team knowledgeable about the disorder is important for the best outcomes but there is much more work to be done. This review will focus on the practical aspects of diagnosing and managing FND.

## Introduction

Functional Neurological Disorder (FND) is one of the most common neurological disorders seen from childhood to the elderly, in almost all clinical settings, and by clinicians from different disciplines, especially neurologists [1]. The last two decades have seen an improved understanding of this disorder that has brought a renewed clinical approach guided by increasingly available evidence. These advances allow clinicians to more comfortably diagnose and manage patients with FND in their pursuit of recovery. This article summarizes practical management highlights to guide clinicians who come across individuals with FND in their clinical practice.

FND is currently defined as a “clinical syndrome with genuinely experienced neurological symptoms which are distressing or impairing, and show dysfunction of the nervous system with variability in performance within a task or between tasks” [2]. FND is associated with high and rising healthcare utilization and costs, similar to other complex neurological disorders but with marked lag in research funding comparatively [3,4]. In addition, there are a growing number of studies showing high rate of disability and distress with FND diagnosis [5] as well as elevated morbidity and mortality especially in the seizure subtype, functional/dissociative seizures (F/DS), also known as psychogenic nonepileptic seizures [[6], [7], [8]]. This is particularly concerning since FND is a challenging condition to recognize, diagnose, and treat. These challenges have led to the increased costs but suboptimal outcomes [9]. In 2013, the DSM 5 update required FND (recognized within the DSM 5 manual as “conversion disorder” or “functional neurological symptom disorder”) be diagnosed using positive “rule in” criteria, abandoning longstanding practice of ruling out all other possible causes, and helping to make some progress with the recognition of the disorder. There is a new dawn for FND with a rapidly growing interest in research into understanding how this disorder happens, educating clinicians and the public to how common it is, and in searching for new evidence-based treatments that can help the myriads of patients who suffer from it. This current perspective will provide a brief background and then focus on practical management of this common, distressing and debilitating disorder.

### Subtypes and terminology

There are multiple subtypes of FND with overlap of symptoms common to the disorder (see Table 1). The most common types are seizure and motor subtypes. Other subtypes include the recently described persistent postural and perceptual dizziness or “3PD” for short and the cognitive subtype as well as other types that have been recognized for a long time including speech, vision, swallowing, bladder, and sensory symptoms [2]. Words matter and for the seizure subtype, agreement on the label remains unsettled and has led to confusion and avoidance of care on the part of patients. The International League Against Epilepsy and the Functional Neurological Disorder Society are working toward adopting a combined label Functional/Dissociative Seizures (F/DS) to not ‘offend’ with words such as ‘psychogenic,’ or cause iatrogenic harm [[10], [11], [12], [13]] with now-rejected terms such as ‘pseudoseizures‘ or ‘hysteria/hysterical.’ FND currently seems to be an acceptable term for patients and clinicians (though not perfect) indicating it as a brain network disorder since evidence for underlying structural abnormalities on testing is generally unremarkable.

**Table 1:** FND subtypes, adapted from Hallett et al., Lancet Neurology, 2022.

FND subtype	Description
Functional seizures	Seizures that resemble epileptic seizures but lack the characteristic electrical discharges. Example of positive signs: Eye closure, prolonged duration, on-and-off pattern, head shaking, asynchronous limb movements.
Functional movement disorders	Includes tremors, dystonia, myoclonus, weakness, and other abnormal movements that are inconsistent with known neurological diseases. Example of positive sings: Movement decreases with distraction, enhances with attention, entrains with exposure to a different movement pattern, Hoover's sign, uneconomic gait, knee buckling.
Persistent perceptual postural dizziness	Chronic dizziness and unsteadiness not explained by vestibular or neurological disorders. Example of positive signs: Non-spinning vertigo present most of the time that may exacerbate and persist with certain movements or complex visual patterns.
Functional cognitive disorder	Cognitive symptoms such as memory problems and concentration difficulties without a clear organic cause. Example of positive signs: Internal inconsistencies in testing, patient provides detailed recount of cognitive deficits.

### Epidemiology, risk factors, and co-morbidities

The incidence of FND in adults is estimated at 10–22/100,000 people and in children, 1–18/100,000 in a recent systematic review using original research on people with FND [14]. The study estimated the prevalence at 80–140/100,000 people with a range of 50–1600/100,000 by combining selected studies. The onset of FND occurs most commonly between adolescence and midlife and almost always reported with higher frequency in females (3:1) except in prepubertal children and elderly where it occurs equally [15]. FND is likely more common in women due to increased incidence of common risk factors including post-traumatic stress disorder (PTSD), depression, anxiety, migraine, and autoimmune disorders, though further research is needed. Neurological and medical comorbidities are common risk factors for FND, and increase the likelihood, but do not serve to confirm a diagnosis, or to suggest the cause of FND. Co-morbid psychiatric disorders such as depression, anxiety, PTSD and personality disorders are often but not always present in FND. Co-morbid neurological subspecialty clinics for epilepsy, multiple sclerosis, Parkinson's disease, and stroke are noted to have approximately 20 ​% of patients with FND [16]. FND is reported commonly after injury such as concussion [17] and frequently co-exist with other complaints including chronic pain, sleep, fatigue, and cognitive complaints [18], all of which are considered risk factors for FND. In children, family adversity and school challenges, including bullying, are more likely among the common predisposing risk factors to developing FND than sexual abuse, which is more common in adults [[19], [20], [21]]. In FND, as in many disorders, incorporating a biopsychosocial model with environmental factors contributing to and perpetuating the disorder is essential.

### Neural and cognitive processing mechanisms

There is a growing volume of scientific articles focused on the potential mechanisms of persistent physical symptoms and FND [22,23]. Most of the studies have been in the motor subtype though studies on functional seizures are emerging. Dr. Mark Hallett re-introduced the prescience of Charcot's terminology “la lesion dynamique” during his Wartenberg lecture at the 2024 American Academy of Neurology meeting, showing the accumulating evidence supporting FND as a disorder within brain networks involving emotion processing, motor control, agency, predictive coding, and interoception, all influenced by biopsychosocial factors [24]. Innovative studies on brain networks responsible for self-agency [25], motor intention and planning [[26], [27], [28]], connectivity between motor planning and emotion regulation [29], altered interoception [30,31], inference and predictive processing [2,22] have been instrumental in moving the field forward. More recent work in this area has shown the relationship between biopsychosocial factors in FND and involvement of the amygdala, orbital frontal cortex, and anterior cingulate [32]. Szaflarski and colleagues' work specifically focused on patients with functional seizures have demonstrated dysfunction involving emotion and motor network pathways [33,34]. Diagnostic biomarkers will be important but have not yet been able to clinically distinguish individuals with FND, however, some promise is beginning to show for group differences compared to other neurological disorders [35].

## Diagnostic phase

There remains broad lack of knowledge about and recognition of FND among clinicians which leads to delay in diagnosis [13,36,37]. In addition, there is lack of comfort and knowledge for many clinicians in how to help patients with persistent physical symptoms [38] such as occur with FND. Even when the diagnosis is made with certainty, it is often not properly documented in medical records [39], coded properly [40] or communicated with other clinicians involved in caring for the patient. Recent efforts have been underway in the FND Society and elsewhere to introduce a formal curriculum into medical and neurological education rather than just consider FND as a differential diagnosis to exclude while testing for every other possible disorder (even unlikely ones) and allowing “experiential learning” to occur. A curriculum should include required medical knowledge, clinical skills and communication as with any other neurological disorder, certainly one as common as FND [41].

Diagnosing FND can be challenging as patients typically arrive in the neurologist's office after several consultations and testing without clear answers, with multiple prescribed medications, and common frequent comorbid symptoms. Trust in the health care system or clinicians for some patients can be quite low and allowing them to vent a little may be helpful to understand the patient. Patients may present urgently in high stake settings where treatment is inevitable and potentially harmful such as in the emergency department or post-operatively. Symptoms of FND are variable and they may cross into multiple subspecialist domains. (i.e., movement disorders, epilepsy, headache).

### Obtaining the history

Approach to the patient with FND should follow the same rules for diagnosing any medical disorder. The history should begin with a nonjudgmental mindset that everyone experiences functional symptoms and to consider a broad differential from the start to best help every patient. Many patients with FND have been told of other diagnostic “possibilities” which are remembered as definitive (ie, hemiplegic migraine, transient ischemic attack) from neurologists or emergency department clinicians leaving FND off the differential for someone else to bring up. Alternatively, patients may feel a lack of concern from well-meaning clinicians when presented with “good news, all tests returned normal” implying no disease and offering no follow up. Putting effort into listening to the patient's concerns and beliefs about their symptoms helps identify readiness for treatment. Due to the high rate of traumatic experiences reported in all people, especially those with FND, using communication that fosters trust and reduces harm has been shown to impact patient engagement and improve outcomes [42]. Trauma-informed care principles should be incorporated in interactions with all patients. These include making patients feel safe, having choice in their care decisions, collaborating with the clinician, developing trust, and feeling that they are empowered in their own care. A detailed review of symptoms may uncover migraine or other chronic pain disorder, cognitive complaints such as ‘brain fog’, fatigue, and other neurological symptoms commonly experienced by patients with FND that may need to be addressed before treatment which requires active participation to be helpful. Identifying the possibility of a functional cognitive disorder, a common mimic of Alzheimer's Disease, should be considered in those presenting mainly with memory/cognitive decline of abrupt onset, at young age and with shorter duration of symptoms. Comorbid sleep disorders and psychiatric comorbidities are also elevated and should be carefully screened for [43]. Many but not all patients identify a precipitating injury or incident and describe a sudden and maximal onset of symptoms right at the start [44]. For paroxysmal FND such as seizure or syncope, some patients report recognizing vulnerable states or triggers that also can be helpful for future intervention. Many patients with FND do not report experiencing “stress” and do not appreciate when the clinician assumes psychiatric disorder or “stress” as the cause. There are many potential pitfalls to avoid in diagnosing FND [45,46].

### Establishing the diagnosis

Since FND is common and overlaps with other neurological disorders, all neurologists should be aware to include it for almost any patient with unexplained neurological symptoms and know how to establish the diagnosis. Using several highly sensitive and specific positive signs in someone with high pretest probability, compatible history, and risk factors makes it likely that the diagnosis is correct, especially from a knowledgeable and skilled neurologist. Awareness of the various subtypes of FND [2] should be basic for all neurologists. The most sensitive and specific positive signs for the two most common subtypes of FND are listed in Table 2 [47]. For example, for functional seizures, eye closure with bilateral side to side movements that are prolonged, in the setting of a negative ictal EEG makes an epileptic seizure very unlikely [48] though does not completely rule out comorbid F/DS and epilepsy. In cases of dual diagnoses, prolonged EEG recording and absence of a history of other seizure types are needed. For functional leg weakness, demonstrating a dragging leg gait, a positive Hoover's sign and improvement when the patient runs, or walks backwards, bypasses overlearned pathways in the brain and can reveal preserved ability utilized by physiotherapists (PT) to aid in treatment. Similarly, entraining a tremor or other movement to a simultaneously performed task at a different rate is another distraction technique to help establish the tremor's etiology as functional. Demonstrating this to patients can help with recovery [16].

**Table 2:** Clinical signs (adapted from Table 2. Aybek and Perez BMJ 2022).

Clinical sign	Sens. %	Spec. %	Comments
Hoover's sign	60–100	86–100	Unilateral leg weakness; and no lesion in opposite SMA or parietal lobe
Hip adductor sign	–	100	Unilateral leg weakness
“Giveway” weakness	20–90	95–100	Absence of joint pain which can cause limb to “giveway” in anyone suffering with pain on movement of limb
Dragging leg gait	20–100	100	Monoplegic/weak leg is externally rotated while dragged
Tremor	89.5	100	Entrainment, pause, or distruption of the tremor with unaffected limb tapping at different rates and amplitudes
Drift w/o pronation	47–93	100	Palms up, wait 10 ​s; mild-mod unilateral UE “giveway” weakness without pronation
Ictal eye closure	34–88	74–100	Geotropic (downward) gaze w/forced eye opening; blinking after rubbing eyelashes during a seizure
Ictal weeping	3.7–37	100	Whimpering or crying during a functional seizure. Confirm that it was not postictal
Pelvic thrusting	1–44	92–100	Consider frontal lobe seizure if movements are stereotyped, occur from EEG proven sleep, are brief, or occur in a cluster
Side to side head/body	25–63	96–100	Convulsive events only
Asynchronous movements	44–96	93–96	Consider frontal lobe seizure if stereotyped, occurs from sleep, is brief, or clusters
Fluctuating course/long dur.	47–88	96–100	–
Sensory loss- midline split			Not reliable, seen with thalamic stroke

Prescribing a medication or ordering a test is unavoidable in certain settings and situations, but it is best to be strategic and minimalistic, if possible, when FND is high in the differential, explaining what is the most likely diagnosis and what is expected from the tests so that patients are not left to worry needlessly. Honest and transparent discussion before patients are sent for testing allows for two-way communication of concerns, and absence of any perceived deception at removing a long held incorrect diagnosis. Overlap with comorbid neurological conditions is not uncommon, and sometimes symptoms appear functional before another disorder clearly emerges, so caution is warranted to overly diagnose FND and to assume all new subsequent neurological symptoms after diagnosis of FND will be functional. For seizures which are transient and therefore more challenging to diagnosis in the office, capturing an ictal convulsive event on a smart phone with expert review may be enough, though video EEG is still considered the gold standard. Not every patient will be able to have episodes captured on video EEG. Laboratories, procedures, or imaging of the brain or spine is often necessary early in presentation especially if there are signs of an accompanying neurological disorder, when predisposing risk factors are absent, or when history and exam are not conclusive. Many neurologists are more concerned about missing an alternate diagnosis when it is much more common to miss a diagnosis of FND [49].

FND requires a multidisciplinary team-based approach to assure proper diagnosis and formulation of contributing factors as readiness for treatment is assessed simultaneously during the diagnostic evaluation. Input from mental health professionals (evaluating psychosocial stressors, psychological traits and psychiatric comorbidities) and rehabilitation specialists (evaluating functional limitations to engage in their treatments) may help inform a comprehensive biopsychosocial assessment. This approach focuses on the entire patient, the entire brain, avoiding the dualistic mindset of brain and mind as two separate entities.

## Management

### From explanation of diagnosis to engagement in treatment

Communication is the most common procedure in medicine and the skill that is critical for explaining what is happening for the patient to understand. If poorly delivered, patients are likely to seek more consultations or feel further stigmatized by the health system and not seek treatment.

As soon as there is reasonable suspicion of an FND diagnosis based on history and exam, this should be mentioned to patients. This early approach can prevent the news of the diagnosis from becoming an unwelcome surprise later and allows patients and loved ones to become accustomed to the usually unfamiliar concept of FND.

Existing communication protocols in FND, most developed specifically for functional/dissociative seizures (F/DS), review specific points that should be shared with patients when the diagnosis is explained for the first time [[50], [51], [52]]. Adding a neuroscience-informed explanation of FND, that takes into account psychological risk factors can assist clinicians in the diagnosis delivery and may help dispense clinicians with the dichotomy of neurobiological and psychological processes [53]. Many patients resist psychological explanations [54] and may feel blamed if only a psychological explanation is provided for their diagnosis. It is important to tell patients what their diagnosis is, and not what it is not. Table 3 lists elements to be considered when delivering a diagnosis of FND, with a sample script for functional weakness.

**Table 3:** Elements to be covered during initial communication of diagnosis of FND with sample script for functional weakness in right lower extremity.

1) *Give a name to the disorder*: “What you have is called functional neurological disorder; in your case, the specific symptom is functional weakness. You may hear that we refer to these symptoms as ‘subtypes’ of FND (i.e., gait, weakness, seizures, etc)”
2) *Explain it is a common diagnosis made in neurology clinics*: “Although you may not have heard about FND before (*if applicable*), this is actually a very common diagnosis. It is actually one of the most common diagnoses made in neurology clinics (*or different setting, if applicable*)”
3) *Reassure patient that FND does not imply ‘faking’ and symptoms are believed to feel involuntary*: “I want to reassure you that your FND symptoms are not fake, or made-up. Although we do not yet know all that happens in the brain that leads to FND, there are elegant studies that show us that in FND, for example, there is a miscommunication between different parts of the brain responsible for generating movement and for creating a sense that a movement you made is yours.”
4) *Show how the diagnosis was made (i.e., explain how the Hoover's sign helped in the diagnosis)*: “In your case we know that your weakness is functional because when I examined you, I was able to demonstrate that your right weak leg was strong if I was asking you to support movement in the left unaffected leg; that shows me that there is strength in the right, weak leg, but you may not be able to move it if, for instance, we are paying too much attention to it. This tells me that the weakness is caused by functional neurological disorder.”
5) *Provide an individualized explanation of the disorder (mechanism and biopsychosocial formulation):* “Functional neurological symptoms happen when our brain, for some reason, starts to misread signals from the body and then creates expectation of how a body part may move or feel. Like with all movements, you are not aware of this while it is happening. For example, in your case, after you injured your right knee and started to experience pain, the brain started to read signals from your right leg differently, with more alert than usual. You also told me that a year before you were undergoing problems with your marriage and were experiencing panic attacks, which probably influenced how sensitive your brain became at reading any signal of body discomfort. This may have created a ‘perfect’ storm for your functional leg weakness to develop. Additionally, you told me your sister had fibromyalgia and chronic fatigue syndrome when you were a teenager, and these disorders share some mechanisms with FND, so it is possible that there is some genetic predisposition.”
6) *Share treatment options/path to recovery*: “There is treatment for FND and many patients recover if they fully participate in treatment. In your case, we will prioritize re-training your brain on how to move your right leg. This will take time and effort, and a professional, such as a physical therapist with experience in FND, will guide you in the process. It is essential that the other diagnosis we discussed, such as your panic disorder, remains well controlled.”

Beyond the content of the discussion, an empathic and caring attitude which uses a trauma-informed care approach is of critical importance in fostering a positive therapeutic relationship. Eliciting concerns about the clinician's explanation of diagnosis so that patients can ask questions for improved understanding is helpful. Patients may not agree with the diagnosis which signals a greater likelihood of them not following the recommended treatment, so allowing them time to come back and rediscuss with other supports is helpful as this is a process that can take time and should not be rushed. In addition to explaining the disorder to the patient, there is often a need to educate others who are involved in helping the patient, including family supports, primary care providers, therapists, other specialists, or roommates.

Physician attitude may be the deciding factor determining whether a patient accepts the diagnosis and engages in treatment or whether they move on to search for new diagnostic explanations, which may further delay treatment [55].

Evidence shows that treatment within 30 days of diagnosis led to marked improvement in outcome in children with F/DS [56]. Motivational interviewing (MI) is a communication approach to facilitate change and it can be applied to the initial discussion following the disclosure of the FND diagnosis to motivate patients to follow treatment recommendations and engage in treatment. MI has been shown to improve treatment adherence in many medical conditions such as substance use disorders, diabetes, HIV [57]. A randomized controlled trial (RCT) showed that adding an MI-informed discussion after a standard explanation of the F/DS diagnosis significantly improved adherence to the recommended treatment at 6 months compared to using the standard communication alone. The MI intervention also significantly improved seizure outcomes and quality of life [58]. These findings emphasize the importance of engaging patients through their internal motivation for change rather than imposing a treatment on them.

It is now accepted that treatment for FND requires involvement of multiple disciplines. Diagnosis is usually confirmed by a neurologist and/or neuropsychiatrist, and treatment is delivered by a combination of disciplines that, depending on each case, may include mental health professionals (of different training background) and rehabilitation professionals (physiotherapists, occupational therapists, speech and language pathologists, cognitive rehabilitation specialists). It is recommended to have other medical professionals involved in the patient's care as well as family, loved ones and community supports (work, school) understand the diagnosis and reinforce treatment participation [59]. Clinicians involved in the patient's care but unfamiliar with FND should be introduced to the concept of FND and offered educational opportunities to learn more about it. Usually the patient's primary care physician (PCP) or the clinician who diagnosed FND should ensure that treatment engagement occurs and help correct any issues that may interfere with treatment participation. Family members skeptical about the diagnosis should openly review their doubts with the team, so that there is an opportunity for them to learn about FND, rather than to create doubt and limit treatment engagement and recovery. This coordination of care should occur with the patient's agreement and their active participation [59]. Working together as a team to reinforce and not contradict the diagnosis and create a biopsychosocial formulation from an in-depth interview that includes targets for treatment based on psychiatric comorbidities, risk factors, personality functioning, cognitive abilities, and coping mechanisms, some of which may need to be addressed as maladaptive is essential [60].

Treatment interventions for FND require time commitment and an embracement of the newly learned and acquired skills, whether these are psychotherapy- or rehabilitation-based. Many factors may signal that a patient is “not ready” for treatment and any identified treatment-interference factors (such as skepticism about the diagnosis, new functional symptoms, avoidance of any discussion of emotional/psychological symptoms, active litigation, prior traumatic experiences with the health care system, active substance use) should be proactively addressed, if feasible [61,62]. For example, in a patient undergoing psychotherapy for FND who misses appointments or does not complete their homework due to interfering headaches, the treating clinician should frankly discuss whether treatment should be delayed until there is improved headache control, or, preferably, review strategies to make headache symptoms more tolerable so that engagement in therapy for F/DS can take place (i.e., limit time completing homework, but still engaging in it for a certain amount of time every day). In a patient with a functional gait who develops weakness during treatment, once etiology of the weakness is confirmed as functional, explaining that both symptoms can improve with a treatment approach that follows the same principles and similar techniques can prevent delay in treatment engagement and accelerate improvement. Patients who want to obtain other medical opinions because they question the diagnosis of FND will fare best if treatment is delayed until they feel convinced enough to pause any new diagnostic exploration.

Any seizure or episode of loss of awareness occurring in a public space will most likely trigger an ambulance call so it is helpful to have a “seizure plan” readily available for those who are symptomatic. This includes trying to keep patients in school, work, or other program, by allowing breaks rather than sending them home or to the hospital when episodes occur. The outpatient clinic is the best place to address these types of issues, preparing the patient and family for the “what if” scenarios.

Professionals offering psychotherapy for patients with F/DS should prepare in advance for the occurrence of seizures in their office or, if the intervention is conducted via telehealth, how to manage a crisis remotely. Recommendations have been published on how to manage such occurrences [63,64].

Offering printed or web-based educational materials (neurosymptoms.org, nonepilepticseizures.com) and connecting patients and loved ones to patient advocacy organizations (fndhope.org) provides both a sense of relief and community support. Many materials offer symptom management tools that patients may find quite helpful and provide an initial sense of mastery over what is initially experienced as an elusive and mysterious disease.

### Evidence-based treatment

The current evidence primarily favors the use of psychological and rehabilitative therapies for the treatment of FND. Which therapy or combination of therapies are offered will not only depend on the phenotypic presentation (i.e., F/DS is usually addressed through skills-based psychotherapy while a functional gait is initially addressed with physical therapy), but also on prior response to treatment (if any) and patient's ability to meaningfully engage.

Psychological therapies remain the most studied form of treatment in FND. Previous meta-analyses show that both cognitive behavioral therapy (CBT)-based and psychodynamic approaches provide medium-sized benefits for physical (functional neurological) symptoms, mental health, well-being, functioning and resource use. While the outcomes are comparable between these two main psychotherapy modalities, psychodynamic trials lacked controlled studies [65,66].

Many manualized psychotherapy approaches have been investigated in FND, some of which have been published [67,68]. These include CBT [69], mindfulness-based psychotherapy [70], prolonged exposure [71], and multi-modality neurobehavior therapy [72]. One explanatory model in these treatment modalities is that F/DS represent a dissociative reaction to hyperarousal [73], which occurs in the context of certain psychological characteristics (avoidance tendencies, somatization, emotion dysregulation) that inform symptom development [[67], [68], [69], [70], [71], [72],^74^]. In the case of CBT and related approaches, the goal of the intervention is to gradually expose patients to their feared or avoided emotion or activities and to change their thinking style regarding symptoms or associated phenomena. Specific treatment protocols offer variations on this theme [75]. Manualized psychotherapies are time-limited (usually 12–15 sessions) and most offer a certain degree of flexibility to adapt to the patient's specific clinical background [75]. Other psychotherapy approaches such as eye movement desensitization and reprocessing (EMDR) [76], hypnotherapy [77], group psychotherapy [78,79] have been explored and documented to be beneficial.

The CODES (Cognitive Behavioral Therapy for Dissociative Seizures) trial is worth mentioning separately given that it is currently the largest randomized controlled trial ever conducted in F/DS. In this multicenter RCT conducted across the UK, 368 adults with F/DS were randomized to either CBT plus standardized medical care (SMC) or SMC alone. The primary outcome measure (monthly seizure frequency at 12 months from randomization) did not differentiate between the two groups; however, many other secondary outcome measures did (including seizure “bothersomeness”, quality of life, functioning, longest seizure free duration) [69]. A post-hoc analysis demonstrated that seizure frequency was better in the CBT group at 6 months post-randomization, which was closer to the end of the intervention [80]. Despite technically a ‘negative trial’ based on the primary outcome measure, there are meaningful clinical benefits from the F/DS-specific CBT utilized in the CODES trial, especially shortly after its conclusion. Additionally, one must consider that the control intervention in this trial was sophisticated clinical care rather than simply a wait-list control or what is usually considered ‘treatment as usual’.

Rehabilitative therapies have become first-line interventions in FND, depending on the specific phenotype. In functional motor disorder (FMD: weakness, gait, abnormal movements), PT should be considered; in functional voice/speech and swallowing phenotypes, speech and language therapy (SLP) should be prioritized. Occupational therapy (OT) can guide adaptations to different functional deficits and can be particularly helpful with sensory symptoms. Consensus recommendations exist for PT, OT and SLP that help guide how to specifically adapt treatment for patients with FND [[81], [82], [83]]. In the case of PT, for example, the emphasis is placed on facilitating automatic or overlearned movements, decreasing focus on the symptom or deficit, encouraging early weight bearing and limiting hands-on support. A specific PT protocol developed in the UK (Physio 4 FMD) was studied in a multi-center RCT and patients were randomized to receive said protocol (called ‘specialist’ PT) versus general (‘non-specialist’) PT. Physical functioning at 12 months from randomization (the primary outcome measure) did not differentiate between the two groups, but there was a higher likelihood of patients endorsing improvement in their symptoms if they had received the ‘specialist’ protocol [84]. The evidence for other rehabilitative therapies is limited and primarily based on case reports.

The increase in evidence-based treatment for FND is encouraging. Yet, given how common the disorder is encountered in clinical practice, use of these approaches in community settings remains rare. Spreading use of evidence-based interventions for FND will require a collaborative training effort so that these therapies can be used outside of a few FND specialized centers or clinics.

### Role of biological therapies

Currently, there is no convincing evidence that pharmacological treatment reduces FND symptoms specifically. There is only one double-blind placebo-controlled study of sertraline in F/DS, which showed that sertraline was not superior to placebo at reducing seizure frequency [85]. The study was probably under-powered given that the active drug group showed a reduction in FDS at treatment end while the placebo group showed an increase, however, this difference was not enough for a statistical difference. Other data comes from open-label design studies [86,87] or isolated case reports. Data from a related diagnostic category, somatic symptom disorder, seems to indicate benefit from antidepressant medications [88,89]. Clinicians ought to be clear what their intended outcome is when prescribing any medication for FND, whether the target is a comorbid psychiatric symptom or a comorbid physical symptom, such as pain, and be upfront that direct impact on FND symptoms is generally not expected from pharmacological interventions. Although there is lack of current evidence for psychopharmacological treatments, occasionally patients may experience symptomatic improvement of their FND symptoms, likely through relief of underlying psychiatric underlying mechanisms.

Both placebo and nocebo responses are common in functional disorders, and therefore a reported history of clinical benefit from medications should be interpreted taking these concepts into consideration and not as an indication of a direct therapeutic drug effect on the functional neurological symptom or as a signal that the diagnosis of FND should be re-considered. Similarly, introduction of a medication should be slow and at low doses to minimize any negative effects in this particularly susceptible population. Experience of negative side effects may, in fact, precipitate new or worsening functional symptoms and such possible outcome needs to be considered before any drug initiation.

Non-invasive brain stimulation techniques such as transcranial magnetic stimulation and transcranial direct current stimulation offer future promise as biological therapies that may be incorporated in the treatment toolkit in FND. To date, evidence of their efficacy remains limited and based on studies of small sample size and variable design using different stimulation protocols and outcome measures [90].

### Practical clinical considerations

Evidence-based treatments may not be readily available to all clinicians. Therefore, professionals who diagnose FND are challenged by limited treatment resources. Also, patient-related factors that signal readiness for treatment, mentioned in the section above (III a), need to be considered when developing an initial treatment plan [61].

Stepped care approaches recommend initial brief psychoeducational interventions, mostly delivered by neurologists, and to only progress to a full treatment course (of psychological or rehabilitation therapy or some combination thereof) if the psychoeducational treatments are not beneficial [91]. Most treatment programs are outpatient-based and delivered over a time-limited period with once to three times per week encounters. In instances where it is logistically possible and clinically indicated, multidisciplinary intensive outpatient or inpatient short-term programs may be considered for those more symptomatic and disabled patients unable to benefit from a regular outpatient-based treatment [[92], [93], [94]]. Addressing interfering comorbid conditions that limit engagement in treatment (active substance use, acute psychosis, acute suicidality, debilitating pain) needs to be prioritized and, if indicated, treatment for FND should be delayed until these conditions are better controlled.

Professionals who offer a different explanation for a functional neurological symptom (i.e., bringing suspicion that a well-documented F/DS “may be” epileptic) and who, at the same time, have a trusting connection with a patient, may do so many times with good intentions. In such circumstances, we recommend educating these professionals and ideally incorporating them into the treatment team to help reinforce the correct diagnosis to increase the likelihood of a good outcome.

There are many times when a definitive diagnosis of FND cannot be firmly established. For example, a description of a seizure semiology suggestive of F/DS but never witnessed or observed on a home video or captured during EEG, would only render a “possible” diagnosis of F/DS [95]; or a described gait impairment may raise suspicion for functional etiology but the diagnosis may not be corroborated without eliciting the gait during a neurological exam or observing it on video. In such circumstances, if further diagnosis confirmation seems unfeasible and would inappropriately delay treatment, clinicians must be honest what their degree of confidence in the diagnosis is and offer treatment recommendations based on the ‘presumed’ diagnosis. Such approaches toward presumed diagnoses are in fact rather common in medical practice.

Many patients with FND may present with chronic and disabling symptoms [96,97] and chronicity has been suggested as a negative prognostic factor in FND [98]. Clinicians need to consider these factors when crafting a treatment plan and set realistic expectations more focused on regaining function and reducing distress than on complete symptom remission. Liaising with patients' long-term community clinicians can be particularly beneficial to maximize recovery in the patient's own setting. Secondary gain must always be considered when outlining a treatment plan. Pending disability claims or litigation based on the functional symptom can create a conflict with a proposed treatment aimed at improving the symptom. In such circumstances, we recommend pausing either treatment or the claim process until either the claim is finalized or treatment is completed, respectively [62]. For patients already receiving disability benefits, a clear conversation on treatment expectations and how it may affect (or not) existing benefits can pave the way to a more realistic outcome. When not yet disabled, accommodations, a slow and gradual return-to-work or return-to-school plan, or a time-limited leave are preferable than an indefinite leave [62].

### Pediatric and other considerations

Children and adolescents with FND require special considerations during assessment and treatment. For example, female predominance in F/DS becomes more apparent after puberty [99]. Risk factors leading to the development of FND are different in the pediatric population, with medical stressors, school and family conflicts being among the more common predisposing and precipitating factors rather than frank abuse or neglect, although these should still be screened for during an initial assessment [100]. Both a mind-body, ‘bottom-up’ [101] and a CBT-informed competing response approach (retraining and control therapy), akin to Tourette's behavioral interventions [102], have been studied in pediatric populations with F/DS and found to be effective. Rehabilitative therapies have been less systematically studied in the pediatric population, but depending on the specific phenotype, FND-informed PT, OT or SLP should be considered [103].

The early incorporation of family and school into psychoeducational and treatment interventions is emphasized as all parties involved need to be aligned with the therapeutic plan. When indicated, family therapy should be considered, including the development of a plan on how to respond to symptoms since support and symptom reinforcement may at times overlap. Similarly, a school plan on how to manage FND symptoms can help avoid unnecessary and alarming responses that can lead to symptom reinforcement.

Pediatric and adult individuals with intellectual disability (ID) present additional challenges in the treatment of FND. Data from F/DS support a higher incidence of comorbid epilepsy, a higher prevalence of sexual abuse, more frequent prolonged episodes and situational triggers in people with F/DS and ID compared to those without ID [104]. There is currently no formal evidence to guide treatment of F/DS in individuals with ID. Based on our experience, in mild ID, some of the existing treatments may be simplified and adapted. In more severe forms of ID, identifying triggers and using contingency management to reduce unwanted behaviors that increase risk of F/DS may be helpful.

## Conclusions and Future directions

Over the last two decades, there has been a deeper understanding of the pathophysiology of FND and more treatment options have been developed. This represents a new era for this previously neglected diagnosis suffered by many patients who often feel misunderstood and stigmatized. Unfortunately, harmful treatment of patients with FND is not completely eradicated [13]. However, these recent developments allow clinicians to more confidently interact with patients with empathy, compassion and non-judgmentally.

It is now recognized that certain practices are counterproductive in the management of patients with FND. For example, telling patients that they do ‘not suffer from a neurological disorder’ and that their problem is ‘beyond the neurologist's area of expertise,’ or that the problem is in ‘their heads,’ implying that symptoms are imagined or willfully fabricated, will likely delay diagnosis acceptance and treatment engagement. The new available evidence and growing resources allow clinicians suspecting FND to, at a minimum, help patients connect with a clinician who can either facilitate further confirmation of the diagnosis and/or help patients navigate access to therapeutic interventions.

There is still much to learn about FND. While cognitive and brain mechanisms underlying FND are better understood, we have limited knowledge about genetic or epigenetic vulnerabilities or neurohumoral biomarkers and how these may be shared (or not) with other neuropsychiatric disorders. Clinicians versed in existing effective treatments for FND are difficult to find, so these therapies need to be more widely disseminated. Additionally, not all patients will respond to the currently available therapies, and more creative approaches need to be developed or modified for chronic or treatment-resistant FND. There are new treatment modalities on the horizon that may provide new hope or could enhance existing therapies. Brain stimulation [3,4], virtual reality-based therapies [105], psychedelics [106] are some examples.

The new Functional Neurological Disorder Society (fndsociety.org) is an international professional organization that offers a multidisciplinary forum for those interested in learning more about FND. It offers educational webinars, courses and conferences and has active working groups across disciplines and different aspects of the disorder. The society particularly welcomes trainees and early career professionals.

Education on FND during the initial formative years of training can be the most valuable investment to further legitimize and help us further understand this disorder which can severely affect many peoples’ lives. While efforts to improve training in FND are underway [41], there is a long path to achieve tangible improvements.

## Author contributions

Dr. Barbara Dworetzky designed and wrote a substantial amount of this manuscript including the abstract/key words, the acknowledgment, the introduction, and the diagnostic phase section. In addition, she edited the entire manuscript, uploaded half of the references in Zotero, and submitted the manuscript, as well as wrote the cover letter, the declaration statement, and the author contributions. She will be the corresponding author as well.

Dr. Gaston Baslet co-designed and wrote a substantial amount of the manuscript including the management and conclusion/future direction sections. In addition, he edited the entire manuscript as well as uploaded half of the references in Zotero.

## Declaration of competing interest

The authors declare the following financial interests/personal relationships which may be considered as potential competing interests: Barbara A. Dworetzky MD reports a relationship with Functional Neurological Disorder Society that includes: board membership. If there are other authors, they declare that they have no known competing financial interests or personal relationships that could have appeared to influence the work reported in this paper.
